# The effect of early life conditions on song traits in male dippers (*Cinclus cinclus*)

**DOI:** 10.1371/journal.pone.0205101

**Published:** 2018-11-14

**Authors:** Lucy Magoolagan, Peter J. Mawby, Flora A. Whitehead, Stuart P. Sharp

**Affiliations:** 1 Lancaster Environment Centre, Lancaster University, Lancaster, United Kingdom; 2 Unaffiliated, Lancaster, United Kingdom; Utrecht University, NETHERLANDS

## Abstract

Song complexity and singing frequency in male birds are shaped by female choice; they signal male quality because song is costly to develop and produce. The timing of song learning and the development of the brain structures involved occur during a period when chicks are exposed to a number of potential stressors. The quality and quantity of song produced by adults may therefore reflect the level of stress experienced during early life, a theory known as the ‘developmental stress hypothesis’. We tested this hypothesis using song recordings and life-history data from an individually marked, long-term study population of wild dippers (*Cinclus cinclus*). The extent to which early life conditions predict adult song traits was investigated using natal brood size as a measure of sibling competition; the rate of provisioning by parents as a proxy for nutritional stress; and residuals of the linear regression between body mass and tarsus length as a measure of nestling condition. The syllable diversity in the songs of adult males was positively correlated with their body condition as nestlings, but there was no significant correlation with either provisioning rate or brood size. Provisioning rate did, however, predict song rate; males in relatively poor condition as nestlings or those raised in smaller broods which were fed more frequently by their parents sang at a higher rate in adulthood. These results support the developmental stress hypothesis and provide some of the first evidence from a wild bird of how the conditions experienced during early life impact adult song. Song traits may therefore provide females with information regarding both the current condition and developmental history of males.

## Introduction

In many bird species, males produce complex songs to defend their territories and attract a mate, and female choice is thought to be a major driver of the evolution of large song repertoires [1,2]. While several studies have shown that singing is not metabolically demanding [3,4], the development of the brain structures necessary for learning complex songs is thought to incur significant energetic costs [5]. Furthermore, time spent learning and performing song is time taken away from other essential activities such as foraging [6] and increases exposure to predation [7]. Females can therefore use song characteristics as measures of male quality [2,8,9], thereby gaining benefits such as proficient paternal care, territory defence, and ‘good genes’ for their offspring [2,10,11].

Songbirds have a specialised auditory-vocal area of the forebrain that is responsible for song learning and production known as the “song system” [7,12]. The development of this region of the brain occurs during the nestling and fledgling period, a time when young birds are most vulnerable to stress [10,12], and it has been shown that damage to the song system can be detrimental to adult song production [13]. Song learning can therefore be an indicator of early ontogeny as individuals that have not encountered stress are more likely to have the resources available to invest in optimal brain development [12]. Adult birds may be constrained in the quality and quantity of the song they are able to produce because the costs of stress experienced during development cannot be compensated for later in life [8,10,14–17]. Originally proposed as the ‘nutritional stress hypothesis’ [12], this idea is now known as the ‘developmental stress hypothesis’ to incorporate all of the different stressors which young birds are exposed to [8].

Many studies have found evidence to support the developmental stress hypothesis by using dietary manipulation or the administration of the stress hormone corticosterone, and testing for negative effects of these treatments on song characteristics in adulthood [18]. Examples include: European starlings (*Sturnus vulgaris*; [8,19]), zebra finches (*Taeniopygia guttata*; [9,20–22]), swamp sparrows (*Melospiza georgiana*; [5]) and song sparrows (*Melospiza melodia*; [23]). However, most research has involved captive birds and there are very few reports of a link between early life conditions and song traits in wild bird populations. One exception is a study of male blue tits (*Cyanistes caeruleus*), in which individuals raised in experimentally enlarged broods were shown to sing shorter strophe bouts than those from reduced broods [17]. Two further studies have used body size measurements as a proxy for early life stress; the length of the innermost primary feather of nestling great reed warblers (*Acrocephalus arundinaceus*) was positively correlated with adult repertoire size [10], whereas adult tarsus length, a known correlate of nestling tarsus, was found to correlate with repertoire size in blue tits [16].

In this paper, we provide one of the first tests of whether a wider range of early life conditions predict adult song traits in a wild bird, the white-throated dipper (hereafter ‘dipper’; *Cinclus cinclus*). Dippers are riverine passerines which sing highly complex songs [24]. It has previously been shown that unpaired males recorded early in the breeding season and assumed to be attempting to attract a mate use a larger number of unique syllables within their songs and sing at a higher rate than paired males [24]. These song characteristics are likely to be favoured by potential mates because female birds are known to prefer exaggerated acoustic traits in males [15,25]. Here, we test the developmental stress hypothesis by investigating whether these key song characteristics, syllable diversity (as a proxy for repertoire size), song versatility and song rate, are influenced by the conditions individuals experience as nestlings.

## Methods

### Study population

Data were collected between January 2014 and June 2016 in an individually marked population of 40–50 pairs of dippers in the River Lune catchment near Sedbergh, Cumbria, UK (54⁰323’N, 2⁰528’W). Individuals in this population are given a unique combination of three plastic colour rings and a standard British Trust for Ornithology metal ring. Each year, all nests in the study site are closely monitored to establish parental identity, the timing and outcome of reproduction and a number of behavioural and life-history traits. Nestlings are ringed and measured when they are nine days old. Measurements include tarsus length (± 0.1 mm) or ‘maximum tarsus’ which is the length of the tarsometatarsus, measured using dial callipers; and body mass (± 0.1 g) taken using electronic scales. Adults are trapped for ringing and measuring using mist nets or hand nets placed over the nest; any first-year birds which were ringed as nestlings and have recruited into the population are also trapped so that they can be measured. Fully grown birds can be sexed according to their wing length (± 0.5 mm), taken from the carpal joint to the end of the longest primary on a closed wing using a capped wing rule [26,27]; for most individuals, sexing could be confirmed from observations of reproductive behaviour (e.g. incubation is carried out by the female only; [28]) and in no cases was there a discrepancy between the two methods.

### Song recording and analysis

Songs from 18 adult males born within the study site were recorded opportunistically from distances of 10-15m using a Sennheiser ME66-K6 shotgun microphone with a Rycote Softie windshield and a standard pistol grip connected to a Marantz PMD661 MKII solid state recorder with a sampling frequency of 44.1 kHz; files were stored in WAV format. It was not possible to measure the true size of an individual’s repertoire of syllables due to the apparent complexity and improvised nature of dipper song; the more recordings made, the more unique syllables were identified. We therefore measured the number of unique syllables produced in a standardised sample of 10 songs, hereafter ‘syllable diversity’, as a proxy for repertoire size. Ten was the minimum number of songs recorded for each individual after those with low signal to noise ratio had been excluded; if more songs were available, then the first 10 were selected so that all songs from any given individual were recorded within the same breeding stage (see below for the categorisation of stages). Spectrograms were produced using Avisoft SASLab Pro, version 5.2.08 [29], with a 512-point fast Fourier transform length and Hamming window function, 75% frame size, a 87.5% window overlap, 86 Hz frequency resolution and 1.45 ms time resolution. All songs were also high pass filtered at 1 kHz to remove low frequency background noise (e.g. the sound of the river); this threshold was chosen because preliminary analyses revealed that a small number of Dipper songs contained elements as low as 1.03 kHz [24].

Syllable diversity was measured using visual and auditory inspections of spectrograms to count the number of unique syllables produced across 10 songs [24]. ‘Versatility’ is another measure of song complexity and was calculated by dividing the number of unique syllables found within one song by the total number of syllables found in that song [30]; this value was then averaged across each of an individual’s 10 songs. ‘Song rate’ was calculated using the number of complete songs produced within a 30-minute period of observation for each individual during which the bird sang at least once, starting at the time when the bird was first observed singing; if more than one observation was available, the first was used. Previous work has shown that these three song traits vary between males recorded at different stages of the reproductive cycle [24], and the same three mutually exclusive breeding stages were classified here: (1) ‘solo’ songs were those of individuals recorded in January or February that had yet to be seen with a partner; (2) ‘pre-breeding’ songs were given by individuals that had been seen with the same partner on at least two occasions foraging, prospecting or nest-building together; and (3) ‘breeding’ songs were those of individuals which had paired up and had nests at the laying, incubation or nestling stage. Insufficient recordings were available to include songs from the same male recorded at different breeding stages. For more details of the song recording and analysis procedures, see [24].

### Provisioning rate

Offspring provisioning rates were measured during 1 h nest observations conducted at least 15 m from the nest and using a portable hide if no natural hiding place was available. In a small number of cases, manual observations were not possible (e.g. if the nest was inside a culvert) and so recordings were made using a Panasonic HC-V160 video camera placed at least 5 m from the nest. For both manual observations and video recordings, the 1 h observation period did not begin until after the first time an adult visited the nest. This minimised the effect of observer or camera presence on provisioning rates, although very few birds ever seemed aware of the observer or camera and in the vast majority of cases offspring provisioning took place within a few minutes of the observer hiding or leaving the camera in place. Provisioning rates were measured by simply counting the total number of feeds made by the two parents during the hour. For each nest, observations were conducted between dawn and noon once in each of the three weeks of the nestling period: on day 2, day 10 or 11, and day 17 or 18.

### Statistical analyses

General or generalised linear models were used to test whether syllable diversity (Gaussian error structure), versatility (Gaussian error structure) and song rate (Poisson error structure) in adulthood were predicted by the conditions experienced during early life. These explanatory variables included natal brood size (as a measure of sibling competition), parental provisioning rate (as a measure of nutritional stress, whether mediated by parental investment or food availability) and body condition when nine days old. The breeding stage of the male at the time of song recording was also fitted to account for previously shown effects [24] and the age of the male was fitted to control for potential changes in song characteristics with age. ‘Brood size’ was recorded as the highest number of nestlings within the individual’s natal nest at any time during the nestling period, but then input into the models as a factor due to limited variation: ‘small’ or ‘large’ according to whether it was above or below the mean (mean brood size = 4.39; mean ± SD small brood size = 3.38 ± 0.74; large brood size = 5.17 ± 0.40). ‘Provisioning rate’ was the mean of the provisioning rates from the three observation periods. ‘Body condition’ was calculated using the residuals from a linear regression of the body mass and tarsus length of nestlings when nine days old; this is a widely used measure of condition (e.g. [31]). ‘Age’ was measured as a factor as the bird’s age at the time of song recording, either ‘first-year’ or ‘adult’. ‘Breeding stage’ was also a factor relating to the time of recording: ‘solo’, ‘pre-breeding’ or ‘breeding’ as described above. Prior to analysis, all variables were centred and standardised to improve interpretation of main effects [32]. Collinearity between explanatory variables was checked using pairwise scatterplots, correlation coefficients and variance inflation factors [33]. All variables were considered because there was no collinearity between them (r < 0.4 in all cases) and variance inflation factors were small (< 3; 31). These five explanatory variables were fitted in the full model along with the interactions between all pairs of early life variables. All models were constructed in R, version 3.2.2 [34], and the full model for each song trait was subjected to the ‘dredge’ function in the package ‘MuMIn’ [35] to rank all sub-models by Akaike's Information Criterion, with the Hurvich and Tsai correction for small sample size (AICc; 34,35). If ΔAICc ≤ 2 between two or more of the most parsimonious models, model averaging was performed using MuMIn. Models were checked for overdispersion and validated by plotting the distribution of the residuals, the residuals versus the fitted values and the residuals versus each of the covariates [36].

## Results

### Syllable diversity

**Fig 1:**
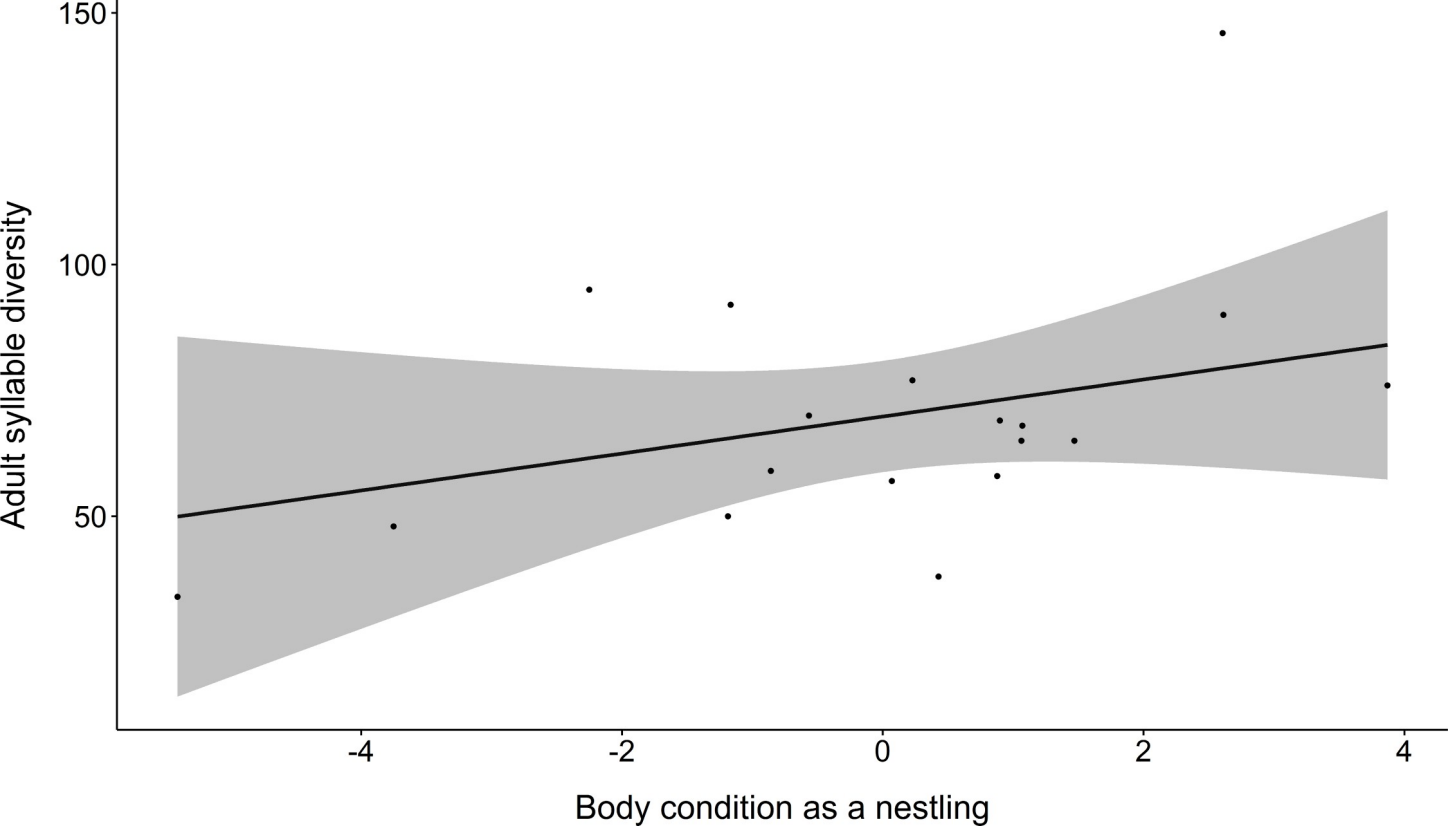
The relationship between nestling body condition and syllable diversity in adult song. The line shows the predicted relationship from averaging of the best fitting general linear models; the raw data are plotted as points. The 95% confidence interval is shown in grey.

The syllable diversity of adult male dippers was predicted by their body condition as nestlings; males in better condition when nine days old produced a greater number of unique syllables within their adult songs (Table 1, Fig 1). No other variables were contained within the best fitting models (Table 1 and S1 Table).

**Table 1:** The best fitting general linear models of the factors associated with adult male syllable diversity: lm(syllable diversity ~ age + body condition + brood size + breeding stage + provisioning rate + body condition: Brood size + body condition: Provisioning rate + brood size: Provisioning rate) Only those models with an AICc value within 2 of that of the best fitting model are shown. For the full list of models see S1 Table.

Model #	(Intercept)	Age	Body condition	Brood size	Breeding stage	Provisioning rate	Body condition x Brood size	Body condition x Provisioning rate	Brood size x Provisioning rate	df	loglik	AICc	Delta	Weight
**1**	69.83		12.03							3	-81.07	169.8	0.00	0.28
**2**	91.00									2	-83.33	171.5	1.61	0.13

### Versatility

Breeding stage was an important predictor of versatility, supporting previous findings; solo males produced songs with the highest versatility and breeding males the lowest (Table 2, S1 Fig). Provisioning rate was also contained within the best fitting models but the effect size was negligible (Table 2). No other variables were present in the best fitting models (Table 2 and S2 Table).

**Table 2:** The best fitting general linear models of the factors associated with adult male versatility: lm(versatility ~ age + body condition + brood size + breeding stage + provisioning rate + body condition: Brood size + body condition: Provisioning rate + brood size: Provisioning rate) Only those models with an AICc value within 2 of that of the best fitting model are shown, apart from the null model which is included by way of comparison. For the full list of models see S2 Table.

Model #	(Intercept)	Age	Body condition	Brood size	Breeding stage	Provisioning rate	Body condition x Brood size	Body condition x Provisioning rate	Brood size x Provisioning rate	df	loglik	AICc	delta	weight
**1**	0.58				+					4	25.35	-39.6	0.00	0.38
**2**	0.57				+	0.02				5	26.54	-38.1	1.54	0.18
**null**	0.51									2	20.13	-35.5	4.15	0.05

### Song rate

**Fig 2:**
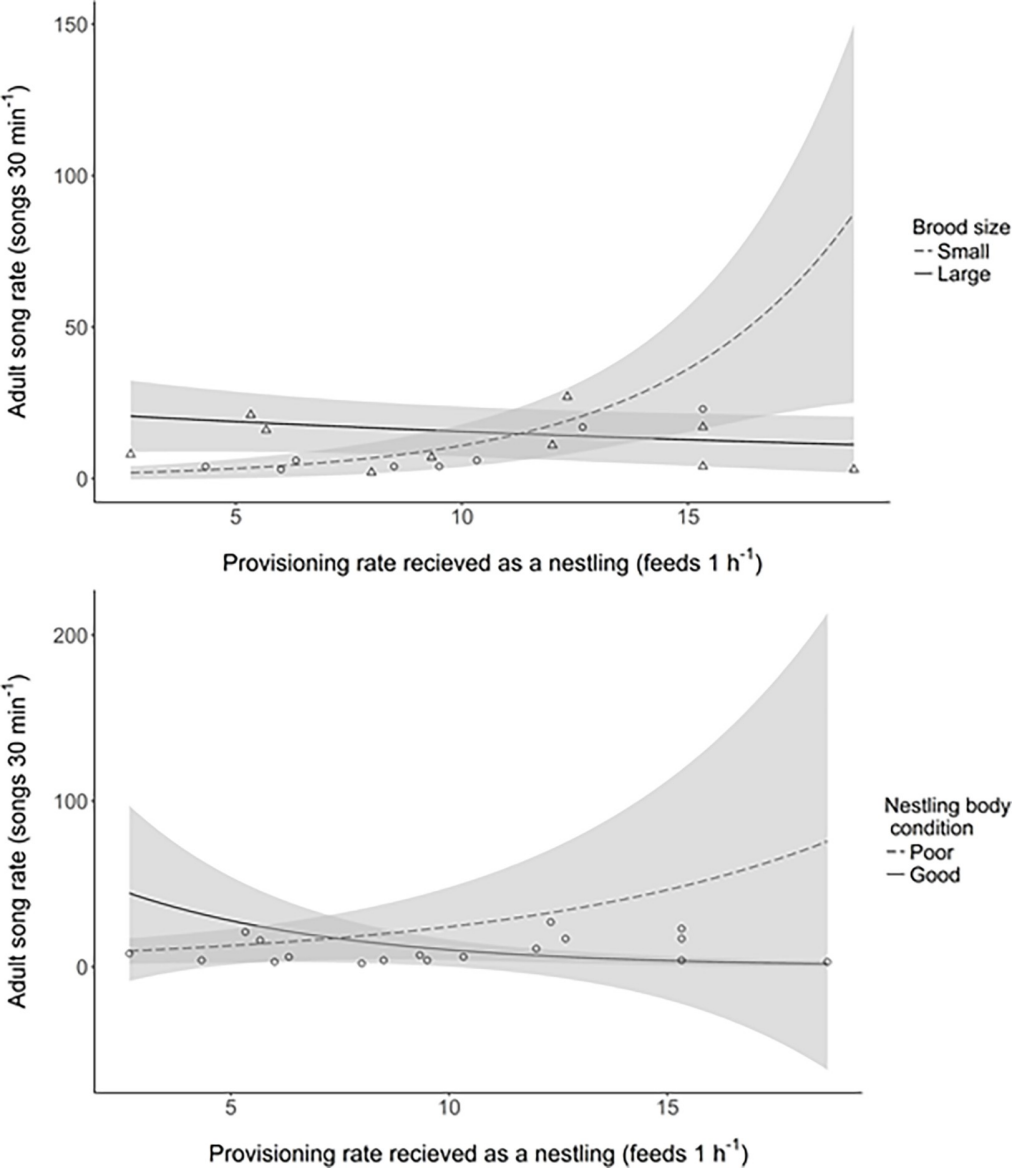
The relationship between the rate at which nestlings were provisioned and their song rate as adults. The lines show the predicted relationships from averaging of the best fitting generalised linear models, plotted for ‘solo’ breeding stage, with the 95% confidence intervals shown in grey. **(a)** The *dashed line* shows the relationship for birds raised in small broods (< 4.39 nestlings, raw data plotted as circles) and the *solid line* shows the same relationship for nestlings in large broods (> 4.39 nestlings, raw data plotted as triangles), both plotted for mean nestling body condition. **(b)** The *solid line* shows the relationship for nestlings in good condition on day 9 (mean condition + 1 SD) and the *dashed line* shows the same relationship for nestlings in poor condition on day 9 (mean condition– 1 SD), both plotted for large broods (>4.39 nestlings); the raw data are plotted as points.

The song rate of males was predicted by the rate at which they were provisioned as nestlings; birds fed at a higher rate by their parents sang at a higher rate as adults (Table 3). However, the best fitting models of song rate also contained the interactions between provisioning rate and both body condition and brood size; the effect of provisioning rate on song rate was greater for individuals in poorer condition and for those raised in small broods (Table 3; Fig 2A and 2B). Song rate also varied with breeding stage, confirming previous findings; solo males sang at a higher rate than pre-breeding and breeding males (Table 3, S2 Fig). No other terms were contained within the best fitting models (Table 3 and S3 Table).

**Table 3:** The best fitting generalised linear models of the factors associated with adult male song rate: glm(song rate ~ age + body condition + brood size + breeding stage + provisioning rate + body condition: Brood size + body condition: Provisioning rate + brood size + provisioning rate, family = “poisson”) Only those models with an AICc value within 2 of that of the best fitting model are shown, apart from the null model which is included by way of comparison. For the full list of models see S3 Table.

Model #	(Intercept)	Age	Body condition	Brood size	Breeding stage	Provisioning rate	Body condition x Brood size	Body condition x Provisioning rate	Brood size x Provisioning rate	df	loglik	AICc	delta	weight
**1**	2.09		-0.09	+		1.16		-0.28	+	6	-59.17	138.0	0.00	0.42
**2**	2.63		-0.25	+	+	1.00		-0.35	+	8	-53.21	138.4	0.45	0.34
**null**	2.32									1	-84.6	171.5	33.5	0

## Discussion

The conditions individuals experience during the early part of their lives have been shown to impact a range of life history variables and phenotypic traits in adulthood [37,38], including behavioural traits such as birdsong [19,39]. Our results demonstrate that song traits in male dippers are correlated with early life conditions and provide some of the first evidence from a wild bird population to support the developmental stress hypothesis [8,12]. This hypothesis proposes that song production and complexity may be compromised by exposure to stress during the nestling period as this is when the brain structures responsible develop [5,8,12]. Nestling body condition, which likely reflects nutritional stress, was positively correlated with syllable diversity, a proxy for repertoire size; this supports the findings of several experimental studies of captive birds in which the quality of nestling diet was manipulated [9,19,21]. The nestling phase is a critical period of growth for birds [40], and individuals in poorer condition at this time are presumably unable to invest as heavily in the development of the brain structures associated with song learning [5,41]. Such birds would be unable to learn and produce as many different syllables, hence syllable diversity may provide females with an honest signal of male quality [2,8,9]. Interestingly, versatility was found to vary with breeding stage but was not correlated with any of the measures of early life conditions. Solo and pre-breeding males sing with higher versatility than breeding males [24], suggesting that individuals are able to vary the use of their repertoires according to context even if the size of their repertoire is constrained by developmental stress. However, further research is needed to explore these ideas, in particular the analysis of songs recorded from the same males at different breeding stages.

The rate at which male dippers were fed as nestlings was an important predictor of their song rate as adults. In contrast to syllable diversity, song rate is usually considered a plastic trait associated with the current condition of an individual, rather than developmental history ([39,42,43], but see [8]). However, it is often reported that developmental stress can cause long-term damage and have a negative effect on adult phenotypes, thereby affecting an individual’s ability to cope in their current environment [38]. For example, zebra finch chicks that were raised on a poor quality diet exhibited increased resting metabolic rates as adults [44]; this leads to an increase in daily food demand, which in turn may increase the time an individual spends foraging and decrease the time spent performing other behaviours such as singing [44]. Individual song sparrows experiencing less stress during development were found to have better body condition as adults [45], and condition directly affects song output because only individuals with sufficient energy reserves are able to sustain high song rates [43].

As with all altricial species, dipper chicks are completely dependent upon their parents for food for the duration of the nestling period [28,46]. An increase in foraging time and the associated decrease in foraging success has been shown to cause reductions in clutch size, brood size, egg mass, nestling mass, growth rates and survival rates [47–49]. Provisioning rate is therefore likely to have a major effect on the growth and development of each individual, and this may be greater in smaller broods where the per capita effect of a change in food availability is higher. Our results show that individuals raised in small broods which were fed more frequently sang at a higher rate as adults, presumably because the additional resources available to these birds as chicks allowed them to maintain better body condition later in life [5,41]. The apparent absence of this effect in larger broods may be due to sibling competition offsetting the benefits of an increase in provisioning rate. Similarly, the positive correlation between provisioning rate and song rate in individuals whose nestling body condition was relatively poor suggests that nutritional stress in early life impacts the condition-dependent investment in singing behaviour of adults. While some studies have suggested that singing is energetically inexpensive [3,4], adult condition has been shown to be positively correlated with song rate in several species [2,43,50]. Our study is the first to provide evidence that early life conditions can influence song rate as well as complexity in wild birds, and further work is now required to investigate the mechanisms behind these relationships.

## Supporting information

S1 Table The general linear models of the factors associated with adult male syllable diversity. The general linear models of the factors associated with syllable diversity: lm(syllable diversity ~ age + body condition + brood size + breeding stage + provisioning rate + body condition: brood size + body condition: provisioning rate + brood size: provisioning rate, family = "gaussian").
(DOCX)

S2 Table The general linear models of the factors associated with adult male versatility. The general linear models of the factors associated with versatility: lm(versatility ~ breeding stage + age + provisioning rate + brood size + body condition + brood: provisioning rate + brood size: body condition + body condition: provisioning rate).
(DOCX)

S1 Fig The relationship between breeding stage and versatility. Versatility of male songs for different breeding stages, predicted for mean provisioning rate (9.87 feeds per hour). Error bars show the 95% confidence intervals.
(TIFF)

S2 Fig The relationship between breeding stage and song rate. Male song rate for different breeding stages, predicted for large broods, mean provisioning rate (9.87 feeds per hour) and mean body condition (-1.23 x 10^−17^). Error bars show the 95% confidence intervals.
(PNG)

S3 Table The generalised linear models of the factors associated with adult male song rate. The generalised linear models for factors associated with song rate: glm(song rate ~ age + body condition + brood size + breeding stage + provisioning rate + body condition: brood size + body condition: provisioning rate + brood size + provisioning rate, family = “poisson”).
(DOCX)
