# Supplementary material for: The effect of early life conditions on song traits in male dippers (Cinclus cinclus)
Source: PLoS One. 2018 Nov 14;13(11):e0205101. doi: 10.1371/journal.pone.0205101 (PMC6235254; doi:10.1371/journal.pone.0205101)
Supplement: S1 Table — The general linear models of the factors associated with syllable diversity: lm(syllable diversity ~ age + body condition + brood size + breeding stage + provisioning rate + body condition: brood size + body condition: provisioning rate + brood size: provisioning rate, family = "gaussian"). (DOCX) [file pone.0205101.s001.docx]

**S1 table.** **The general linear models of the factors associated with adult male syllable diversity**.

The general linear models of the factors associated with syllable diversity: lm(syllable diversity ~ age + body condition + brood size + breeding stage + provisioning rate + body condition : brood size + body condition : provisioning rate + brood size : provisioning rate, family = "gaussian").

| Model # | (Intercept) | Age | Body condition | Brood size | Breeding stage | Provisioning rate | Body condition: Brood size | Body condition: Provisioning rate | Brood size: Provisioning rate | df | logLik | AICc | delta | weight |
| --- | --- | --- | --- | --- | --- | --- | --- | --- | --- | --- | --- | --- | --- | --- |
| 3 | 69.83 |  | 12.03 |  |  |  |  |  |  | 3 | -81.1 | 169.8 | 0 | 0.28 |
| 1 | 69.83 |  |  |  |  |  |  |  |  | 2 | -83.3 | 171.5 | 1.6 | 0.13 |
| 9 | 91 |  |  |  | + |  |  |  |  | 4 | -80.9 | 172.8 | 3 | 0.06 |
| 4 | 71.95 | + | 12.57 |  |  |  |  |  |  | 4 | -80.9 | 173 | 3.1 | 0.06 |
| 19 | 69.83 |  | 12.31 |  |  | -1.02 |  |  |  | 4 | -81 | 173.2 | 3.3 | 0.05 |
| 7 | 69.28 |  | 12.32 | + |  |  |  |  |  | 4 | -81.1 | 173.2 | 3.4 | 0.05 |
| 5 | 76.75 |  |  | + |  |  |  |  |  | 3 | -82.8 | 173.2 | 3.4 | 0.05 |
| 11 | 87.41 |  | 9.59 |  | + |  |  |  |  | 5 | -79.1 | 173.3 | 3.5 | 0.05 |
| 17 | 69.83 |  |  |  |  | 2.33 |  |  |  | 3 | -83.3 | 174.2 | 4.4 | 0.03 |
| 151 | 69.14 |  | 17.65 | + |  | 18.61 |  |  | + | 6 | -77.4 | 174.4 | 4.5 | 0.03 |
| 2 | 70 | + |  |  |  |  |  |  |  | 3 | -83.3 | 174.4 | 4.5 | 0.03 |
| 10 | 91 | + |  |  | + |  |  |  |  | 5 | -80.2 | 175.4 | 5.6 | 0.02 |
| 39 | 61.11 |  | 25.77 | + |  |  | + |  |  | 5 | -80.2 | 175.4 | 5.6 | 0.02 |
| 13 | 96.56 |  |  | + | + |  |  |  |  | 5 | -80.3 | 175.5 | 5.7 | 0.02 |
| 21 | 77.32 |  |  | + |  | 3.39 |  |  |  | 4 | -82.6 | 176.2 | 6.4 | 0.01 |
| 6 | 78.32 | + |  | + |  |  |  |  |  | 4 | -82.7 | 176.5 | 6.7 | 0.01 |
| 83 | 68.92 |  | 13.79 |  |  | -1.19 |  | 3.56 |  | 5 | -80.8 | 176.6 | 6.7 | 0.01 |
| 25 | 91.18 |  |  |  | + | -0.66 |  |  |  | 5 | -80.9 | 176.7 | 6.9 | 0.01 |
| 20 | 72.25 | + | 13.13 |  |  | -1.74 |  |  |  | 5 | -80.9 | 176.8 | 6.9 | 0.01 |
| 149 | 80.03 |  |  | + |  | 19.65 |  |  | + | 5 | -80.9 | 176.8 | 7 | 0.01 |
| 8 | 71.77 | + | 12.65 | + |  |  |  |  |  | 5 | -80.9 | 176.9 | 7 | 0.01 |
| 23 | 68.6 |  | 13.04 | + |  | -1.4 |  |  |  | 5 | -81 | 177.1 | 7.2 | 0.01 |
| 12 | 87.83 | + | 8.48 |  | + |  |  |  |  | 6 | -79 | 177.6 | 7.7 | 0.01 |
| 18 | 69.71 | + |  |  |  | 2.36 |  |  |  | 4 | -83.3 | 177.6 | 7.7 | 0.01 |
| 27 | 88 |  | 10.24 |  | + | -2.95 |  |  |  | 6 | -79 | 177.6 | 7.8 | 0.01 |
| 15 | 88.17 |  | 9.22 | + | + |  |  |  |  | 6 | -79.1 | 177.9 | 8.1 | 0 |
| 183 | 65.14 |  | 24.15 | + |  | 18.23 | + |  | + | 7 | -77 | 179.3 | 9.4 | 0 |
| 14 | 95.36 | + |  | + | + |  |  |  |  | 6 | -79.8 | 179.3 | 9.5 | 0 |
| 215 | 69.71 |  | 17.09 | + |  | 21.26 |  | -3.32 | + | 7 | -77.1 | 179.4 | 9.6 | 0 |
| 152 | 68 | + | 17.47 | + |  | 19.6 |  |  | + | 7 | -77.3 | 179.8 | 10 | 0 |
| 26 | 91.06 | + |  |  | + | -0.21 |  |  |  | 6 | -80.2 | 180 | 10.2 | 0 |
| 55 | 61.21 |  | 25.73 | + |  | 0.47 | + |  |  | 6 | -80.2 | 180.1 | 10.2 | 0 |
| 40 | 60.74 | + | 25.95 | + |  |  | + |  |  | 6 | -80.2 | 180.1 | 10.2 | 0 |
| 159 | 78.7 |  | 16.5 | + | + | 19.28 |  |  | + | 8 | -74 | 180.1 | 10.2 | 0 |
| 22 | 78.43 | + |  | + |  | 3.24 |  |  |  | 5 | -82.6 | 180.1 | 10.3 | 0 |
| 29 | 96.52 |  |  | + | + | 0.37 |  |  |  | 6 | -80.3 | 180.2 | 10.3 | 0 |
| 84 | 70.9 | + | 14.23 |  |  | -1.72 |  | 3.11 |  | 6 | -80.7 | 181 | 11.2 | 0 |
| 150 | 77.5 | + |  | + |  | 21.61 |  |  | + | 6 | -80.8 | 181.2 | 11.4 | 0 |
| 87 | 68.08 |  | 14.28 | + |  | -1.44 |  | 3.52 |  | 6 | -80.8 | 181.2 | 11.4 | 0 |
| 24 | 71.12 | + | 13.78 | + |  | -2.07 |  |  |  | 6 | -80.9 | 181.4 | 11.5 | 0 |
| 157 | 94.02 |  |  | + | + | 18 |  |  | + | 7 | -78.1 | 181.4 | 11.6 | 0 |
| 47 | 79.93 |  | 22.02 | + | + |  | + |  |  | 7 | -78.4 | 182 | 12.1 | 0 |
| 28 | 88.27 | + | 9.18 |  | + | -2.51 |  |  |  | 7 | -78.8 | 182.9 | 13 | 0 |
| 91 | 86.26 |  | 11.31 |  | + | -3.14 |  | 2.46 |  | 7 | -78.8 | 182.9 | 13.1 | 0 |
| 16 | 88.28 | + | 8.27 | + | + |  |  |  |  | 7 | -79 | 183.1 | 13.3 | 0 |
| 31 | 87.22 |  | 10.68 | + | + | -3.17 |  |  |  | 7 | -79 | 183.2 | 13.3 | 0 |
| 158 | 90.73 | + |  | + | + | 24.15 |  |  | + | 8 | -76 | 184 | 14.2 | 0 |
| 30 | 95.29 | + |  | + | + | 0.51 |  |  |  | 7 | -79.8 | 184.9 | 15 | 0 |
| 160 | 78.04 | + | 14.53 | + | + | 23.78 |  |  | + | 9 | -72.3 | 185 | 15.2 | 0 |
| 247 | 65.23 |  | 24.41 | + |  | 21.29 | + | -3.91 | + | 8 | -76.7 | 185.4 | 15.5 | 0 |
| 119 | 61.49 |  | 25.45 | + |  | 0.29 | + | 2.13 |  | 7 | -80.1 | 185.4 | 15.6 | 0 |
| 184 | 60.05 | + | 26.71 | + |  | 20.79 | + |  | + | 8 | -76.7 | 185.5 | 15.6 | 0 |
| 56 | 60.57 | + | 26.05 | + |  | 0.64 | + |  |  | 7 | -80.2 | 185.6 | 15.8 | 0 |
| 216 | 68.7 | + | 16.94 | + |  | 22.05 |  | -3.25 | + | 8 | -77.1 | 186.2 | 16.3 | 0 |
| 88 | 70.1 | + | 14.69 | + |  | -1.96 |  | 3.07 |  | 7 | -80.7 | 186.6 | 16.7 | 0 |
| 48 | 76.09 | + | 26.16 | + | + |  | + |  |  | 8 | -77.5 | 187 | 17.2 | 0 |
| 223 | 79.8 |  | 15.85 | + | + | 21.52 |  | -3.17 | + | 9 | -73.7 | 187.9 | 18.1 | 0 |
| 191 | 81.09 |  | 12.13 | + | + | 20.32 | + |  | + | 9 | -73.9 | 188.3 | 18.5 | 0 |
| 63 | 80.07 |  | 21.81 | + | + | -0.97 | + |  |  | 8 | -78.4 | 188.7 | 18.9 | 0 |
| 92 | 86.27 | + | 10.26 |  | + | -2.67 |  | 2.9 |  | 8 | -78.7 | 189.3 | 19.5 | 0 |
| 32 | 87.45 | + | 9.65 | + | + | -2.75 |  |  |  | 8 | -78.8 | 189.7 | 19.8 | 0 |
| 95 | 85.77 |  | 11.59 | + | + | -3.29 |  | 2.43 |  | 8 | -78.8 | 189.7 | 19.8 | 0 |
| 120 | 60.49 | + | 25.95 | + |  | 0.55 | + | 2.19 |  | 8 | -80.1 | 192.2 | 22.4 | 0 |
| 248 | 60.12 | + | 26.98 | + |  | 23.87 | + | -3.93 | + | 9 | -76.4 | 193.3 | 23.4 | 0 |
| 224 | 79.05 | + | 13.98 | + | + | 25.72 |  | -2.85 | + | 10 | -71.9 | 195.3 | 25.5 | 0 |
| 64 | 75.5 | + | 26.93 | + | + | 1.84 | + |  |  | 9 | -77.5 | 195.4 | 25.6 | 0 |
| 192 | 76.33 | + | 17.37 | + | + | 23.49 | + |  | + | 10 | -72.2 | 195.8 | 26 | 0 |
| 127 | 80.04 |  | 21.58 | + | + | -1.08 | + | 0.5 |  | 9 | -78.4 | 197.2 | 27.4 | 0 |
| 96 | 85.77 | + | 10.55 | + | + | -2.81 |  | 2.87 |  | 9 | -78.7 | 197.8 | 28 | 0 |
| 255 | 81.32 |  | 12.93 | + | + | 21.99 | + | -2.83 | + | 10 | -73.7 | 198.7 | 28.9 | 0 |
| 128 | 75.5 | + | 26.9 | + | + | 1.82 | + | 0.07 |  | 10 | -77.5 | 206.4 | 36.5 | 0 |
| 256 | 76.44 | + | 18.5 | + | + | 25.59 | + | -3.37 | + | 11 | -71.8 | 209.5 | 39.7 | 0 |
