# Supplementary material for: The effect of early life conditions on song traits in male dippers (Cinclus cinclus)
Source: PLoS One. 2018 Nov 14;13(11):e0205101. doi: 10.1371/journal.pone.0205101 (PMC6235254; doi:10.1371/journal.pone.0205101)
Supplement: S2 Table — The general linear models of the factors associated with versatility: lm(versatility ~ breeding stage + age + provisioning rate + brood size + body condition + brood: provisioning rate + brood size: body condition + body condition: provisioning rate). (DOCX) [file pone.0205101.s002.docx]

**S2 table. The general linear models of the factors associated with adult male versatility**.

The general linear models of the factors associated with versatility: lm(versatility ~ breeding stage + age + provisioning rate + brood size + body condition + brood : provisioning rate + brood size : body condition + body condition : provisioning rate).

|  | (Intercept) | Age | Body condition | Brood size | Breeding stage | Provisioning rate | Body condition: Brood size | Body condition: Provisioning rate | Brood size: Provisioning rate | df | logLik | AICc | delta | weight |
| --- | --- | --- | --- | --- | --- | --- | --- | --- | --- | --- | --- | --- | --- | --- |
| 9 | 0.58 |  |  |  | + |  |  |  |  | 4 | 25.3 | -39.6 | 0 | 0.38 |
| 25 | 0.57 |  |  |  | + | 0.02 |  |  |  | 5 | 26.5 | -38.1 | 1.5 | 0.18 |
| 13 | 0.57 |  |  | + | + |  |  |  |  | 5 | 25.7 | -36.4 | 3.2 | 0.08 |
| 11 | 0.58 |  | 0 |  | + |  |  |  |  | 5 | 25.4 | -35.8 | 3.8 | 0.06 |
| 10 | 0.58 | + |  |  | + |  |  |  |  | 5 | 25.4 | -35.7 | 3.9 | 0.05 |
| 1 | 0.51 |  |  |  |  |  |  |  |  | 2 | 20.1 | -35.5 | 4.2 | 0.05 |
| 29 | 0.57 |  |  | + | + | 0.02 |  |  |  | 6 | 26.7 | -33.9 | 5.8 | 0.02 |
| 26 | 0.57 | + |  |  | + | 0.02 |  |  |  | 6 | 26.6 | -33.5 | 6.1 | 0.02 |
| 27 | 0.57 |  | 0 |  | + | 0.02 |  |  |  | 6 | 26.5 | -33.4 | 6.2 | 0.02 |
| 17 | 0.51 |  |  |  |  | 0.02 |  |  |  | 3 | 20.5 | -33.3 | 6.3 | 0.02 |
| 2 | 0.52 | + |  |  |  |  |  |  |  | 3 | 20.3 | -32.8 | 6.8 | 0.01 |
| 5 | 0.5 |  |  | + |  |  |  |  |  | 3 | 20.3 | -32.8 | 6.8 | 0.01 |
| 15 | 0.55 |  | 0.02 | + | + |  |  |  |  | 6 | 26.2 | -32.7 | 6.9 | 0.01 |
| 3 | 0.51 |  | 0.01 |  |  |  |  |  |  | 3 | 20.2 | -32.7 | 7 | 0.01 |
| 47 | 0.59 |  | -0.05 | + | + |  | + |  |  | 7 | 28.6 | -32 | 7.7 | 0.01 |
| 14 | 0.57 | + |  | + | + |  |  |  |  | 6 | 25.8 | -31.9 | 7.7 | 0.01 |
| 39 | 0.54 |  | -0.06 | + |  |  | + |  |  | 5 | 23.1 | -31.2 | 8.5 | 0.01 |
| 12 | 0.58 | + | 0 |  | + |  |  |  |  | 6 | 25.4 | -31.1 | 8.5 | 0.01 |
| 183 | 0.55 |  | -0.07 | + |  | 0.07 | + |  | + | 7 | 27.7 | -30.3 | 9.3 | 0 |
| 18 | 0.52 | + |  |  |  | 0.02 |  |  |  | 4 | 20.6 | -30.1 | 9.5 | 0 |
| 40 | 0.59 | + | -0.09 | + |  |  | + |  |  | 6 | 24.9 | -30.1 | 9.5 | 0 |
| 21 | 0.5 |  |  | + |  | 0.02 |  |  |  | 4 | 20.6 | -30.1 | 9.5 | 0 |
| 119 | 0.54 |  | -0.07 | + |  | 0 | + | 0.04 |  | 7 | 27.6 | -30.1 | 9.5 | 0 |
| 7 | 0.49 |  | 0.02 | + |  |  |  |  |  | 4 | 20.5 | -30 | 9.6 | 0 |
| 19 | 0.51 |  | 0 |  |  | 0.02 |  |  |  | 4 | 20.5 | -30 | 9.6 | 0 |
| 4 | 0.52 | + | 0.01 |  |  |  |  |  |  | 4 | 20.4 | -29.7 | 9.9 | 0 |
| 83 | 0.5 |  | 0.02 |  |  | 0.01 |  | 0.03 |  | 5 | 22.3 | -29.7 | 10 | 0 |
| 6 | 0.51 | + |  | + |  |  |  |  |  | 4 | 20.4 | -29.7 | 10 | 0 |
| 91 | 0.56 |  | 0.01 |  | + | 0.02 |  | 0.02 |  | 7 | 27.3 | -29.4 | 10.2 | 0 |
| 149 | 0.51 |  |  | + |  | 0.06 |  |  | + | 5 | 22 | -28.9 | 10.7 | 0 |
| 247 | 0.55 |  | -0.07 | + |  | 0.04 | + | 0.03 | + | 8 | 30.3 | -28.6 | 11 | 0 |
| 31 | 0.56 |  | 0.01 | + | + | 0.02 |  |  |  | 7 | 26.8 | -28.5 | 11.1 | 0 |
| 120 | 0.59 | + | -0.09 | + |  | -0.01 | + | 0.04 |  | 8 | 30.2 | -28.5 | 11.2 | 0 |
| 30 | 0.57 | + |  | + | + | 0.02 |  |  |  | 7 | 26.8 | -28.5 | 11.2 | 0 |
| 157 | 0.57 |  |  | + | + | 0.03 |  |  | + | 7 | 26.8 | -28.3 | 11.3 | 0 |
| 28 | 0.58 | + | 0 |  | + | 0.02 |  |  |  | 7 | 26.6 | -27.9 | 11.7 | 0 |
| 16 | 0.55 | + | 0.02 | + | + |  |  |  |  | 7 | 26.2 | -27.1 | 12.5 | 0 |
| 184 | 0.59 | + | -0.08 | + |  | 0.05 | + |  | + | 8 | 29.3 | -26.7 | 13 | 0 |
| 55 | 0.54 |  | -0.06 | + |  | 0 | + |  |  | 6 | 23.1 | -26.5 | 13.1 | 0 |
| 23 | 0.5 |  | 0.01 | + |  | 0.01 |  |  |  | 5 | 20.7 | -26.4 | 13.2 | 0 |
| 8 | 0.5 | + | 0.02 | + |  |  |  |  |  | 5 | 20.7 | -26.4 | 13.2 | 0 |
| 22 | 0.51 | + |  | + |  | 0.01 |  |  |  | 5 | 20.7 | -26.3 | 13.3 | 0 |
| 20 | 0.52 | + | 0 |  |  | 0.01 |  |  |  | 5 | 20.6 | -26.3 | 13.4 | 0 |
| 48 | 0.6 | + | -0.06 | + | + |  | + |  |  | 8 | 29.1 | -26.2 | 13.4 | 0 |
| 63 | 0.59 |  | -0.04 | + | + | 0.01 | + |  |  | 8 | 28.7 | -25.4 | 14.2 | 0 |
| 87 | 0.49 |  | 0.02 | + |  | 0.01 |  | 0.03 |  | 6 | 22.5 | -25.3 | 14.3 | 0 |
| 151 | 0.5 |  | 0.02 | + |  | 0.06 |  |  | + | 6 | 22.4 | -25.2 | 14.4 | 0 |
| 56 | 0.59 | + | -0.09 | + |  | -0.01 | + |  |  | 7 | 25.1 | -25 | 14.6 | 0 |
| 84 | 0.5 | + | 0.02 |  |  | 0.01 |  | 0.03 |  | 6 | 22.3 | -25 | 14.6 | 0 |
| 248 | 0.59 | + | -0.09 | + |  | 0.03 | + | 0.03 | + | 9 | 32.5 | -24.5 | 15.1 | 0 |
| 150 | 0.51 | + |  | + |  | 0.07 |  |  | + | 6 | 22 | -24.3 | 15.3 | 0 |
| 95 | 0.55 |  | 0.01 | + | + | 0.02 |  | 0.02 |  | 8 | 27.5 | -23.1 | 16.5 | 0 |
| 127 | 0.59 |  | -0.06 | + | + | 0 | + | 0.03 |  | 9 | 31.7 | -22.9 | 16.7 | 0 |
| 92 | 0.56 | + | 0 |  | + | 0.02 |  | 0.02 |  | 8 | 27.4 | -22.8 | 16.8 | 0 |
| 24 | 0.5 | + | 0.01 | + |  | 0.01 |  |  |  | 6 | 20.8 | -21.9 | 17.7 | 0 |
| 159 | 0.56 |  | 0.01 | + | + | 0.03 |  |  | + | 8 | 26.9 | -21.8 | 17.8 | 0 |
| 158 | 0.56 | + |  | + | + | 0.03 |  |  | + | 8 | 26.9 | -21.8 | 17.8 | 0 |
| 32 | 0.56 | + | 0.01 | + | + | 0.02 |  |  |  | 8 | 26.9 | -21.8 | 17.8 | 0 |
| 215 | 0.49 |  | 0.03 | + |  | 0.04 |  | 0.02 | + | 7 | 23.1 | -21 | 18.6 | 0 |
| 191 | 0.6 |  | -0.06 | + | + | 0.04 | + |  | + | 9 | 30.3 | -20.2 | 19.4 | 0 |
| 88 | 0.49 | + | 0.02 | + |  | 0.01 |  | 0.03 |  | 7 | 22.5 | -19.7 | 19.9 | 0 |
| 152 | 0.49 | + | 0.02 | + |  | 0.06 |  |  | + | 7 | 22.4 | -19.6 | 20 | 0 |
| 64 | 0.6 | + | -0.05 | + | + | 0 | + |  |  | 9 | 29.1 | -17.8 | 21.8 | 0 |
| 96 | 0.55 | + | 0.01 | + | + | 0.02 |  | 0.02 |  | 9 | 27.7 | -14.8 | 24.8 | 0 |
| 223 | 0.55 |  | 0.01 | + | + | 0.02 |  | 0.02 | + | 9 | 27.6 | -14.6 | 25 | 0 |
| 216 | 0.49 | + | 0.03 | + |  | 0.05 |  | 0.02 | + | 8 | 23.1 | -14.3 | 25.3 | 0 |
| 255 | 0.59 |  | -0.07 | + | + | 0.03 | + | 0.03 | + | 10 | 32.8 | -14.2 | 25.4 | 0 |
| 128 | 0.6 | + | -0.07 | + | + | 0 | + | 0.03 |  | 10 | 32.5 | -13.6 | 26 | 0 |
| 160 | 0.56 | + | 0.01 | + | + | 0.03 |  |  | + | 9 | 27 | -13.5 | 26.1 | 0 |
| 192 | 0.6 | + | -0.07 | + | + | 0.04 | + |  | + | 10 | 30.8 | -10.2 | 29.4 | 0 |
| 224 | 0.55 | + | 0.01 | + | + | 0.02 |  | 0.02 | + | 10 | 27.7 | -3.9 | 35.7 | 0 |
| 256 | 0.6 | + | -0.08 | + | + | 0.02 | + | 0.03 | + | 11 | 33.6 | -1.3 | 38.3 | 0 |
