# Supplementary figures and images for: The effect of early life conditions on song traits in male dippers (Cinclus cinclus)
Source: PLoS One. 2018 Nov 14;13(11):e0205101. doi: 10.1371/journal.pone.0205101 (PMC6235254; doi:10.1371/journal.pone.0205101)

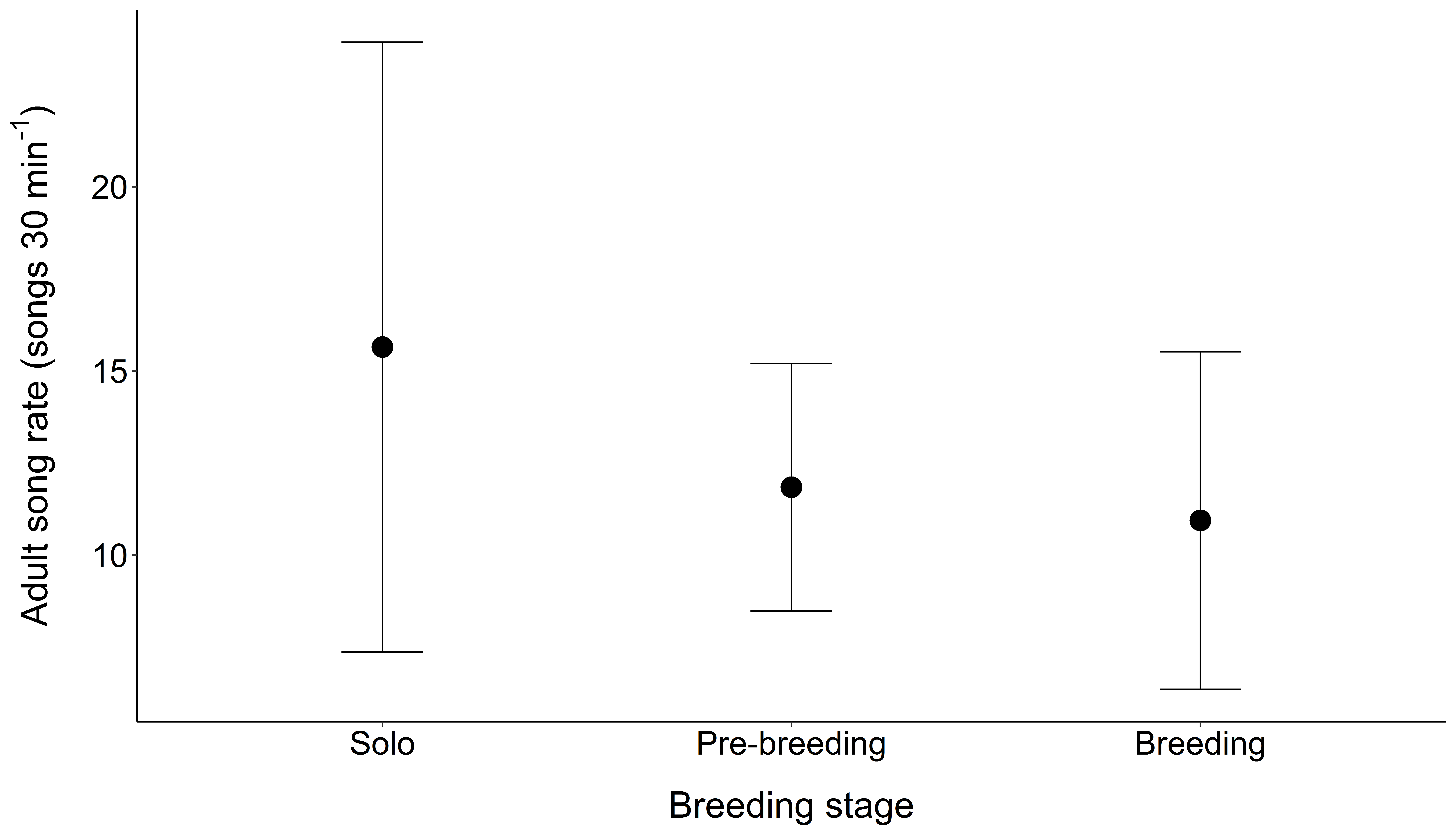

Supplement: S2 Fig — Male song rate for different breeding stages, predicted for large broods, mean provisioning rate (9.87 feeds per hour) and mean body condition (-1.23 x 10^−17^). Error bars show the 95% confidence intervals. (PNG) [file pone.0205101.s004.png]
