# Supplementary material for: The effect of early life conditions on song traits in male dippers (Cinclus cinclus)
Source: PLoS One. 2018 Nov 14;13(11):e0205101. doi: 10.1371/journal.pone.0205101 (PMC6235254; doi:10.1371/journal.pone.0205101)
Supplement: S3 Table — The generalised linear models for factors associated with song rate: glm(song rate ~ age + body condition + brood size + breeding stage + provisioning rate + body condition: brood size + body condition: provisioning rate + brood size + provisioning rate, family = “poisson”). (DOCX) [file pone.0205101.s005.docx]

**S3 table.** **The generalised linear models of the factors associated with adult male song rate**.

The generalised linear models for factors associated with song rate: glm(song rate ~ age + body condition + brood size + breeding stage + provisioning rate + body condition : brood size + body condition : provisioning rate + brood size + provisioning rate, family = “poisson”).

|  | (Intercept) | Age | Body condition | Brood size | Breeding stage | Provisioning rate | Body condition: Brood size | Body condition: Provisioning rate | Brood size: Provisioning rate | df | logLik | AICc | delta | weight |
| --- | --- | --- | --- | --- | --- | --- | --- | --- | --- | --- | --- | --- | --- | --- |
| 215 | 2.09 |  | -0.09 | + |  | 1.16 |  | -0.28 | + | 6 | -59.2 | 138 | 0 | 0.43 |
| 223 | 2.62 |  | -0.25 | + | + | 1 |  | -0.35 | + | 8 | -53.2 | 138.4 | 0.4 | 0.34 |
| 149 | 2.03 |  |  | + |  | 0.9 |  |  | + | 4 | -65.1 | 141.3 | 3.3 | 0.08 |
| 216 | 2.15 | + | -0.08 | + |  | 1.14 |  | -0.3 | + | 7 | -58.8 | 142.9 | 4.9 | 0.04 |
| 247 | 2.06 |  | -0.04 | + |  | 1.15 | + | -0.29 | + | 7 | -59.1 | 143.4 | 5.4 | 0.03 |
| 157 | 2.32 |  |  | + | + | 0.73 |  |  | + | 6 | -62.3 | 144.2 | 6.2 | 0.02 |
| 151 | 2.07 |  | -0.07 | + |  | 0.91 |  |  | + | 5 | -64.9 | 144.7 | 6.7 | 0.01 |
| 150 | 2.04 | + |  | + |  | 0.89 |  |  | + | 5 | -65.1 | 145.2 | 7.2 | 0.01 |
| 255 | 2.51 |  | 0 | + | + | 0.96 | + | -0.39 | + | 9 | -52.6 | 145.8 | 7.8 | 0.01 |
| 224 | 2.62 | + | -0.27 | + | + | 1.01 |  | -0.34 | + | 9 | -53 | 146.5 | 8.5 | 0.01 |
| 159 | 2.5 |  | -0.19 | + | + | 0.74 |  |  | + | 7 | -60.9 | 147 | 9 | 0 |
| 158 | 2.29 | + |  | + | + | 0.76 |  |  | + | 7 | -61.9 | 149 | 11 | 0 |
| 183 | 2.12 |  | -0.16 | + |  | 0.93 | + |  | + | 6 | -64.7 | 149 | 11.1 | 0 |
| 27 | 2.83 |  | -0.34 |  | + | 0.26 |  |  |  | 5 | -67 | 149.1 | 11.1 | 0 |
| 91 | 2.95 |  | -0.41 |  | + | 0.26 |  | -0.17 |  | 6 | -64.8 | 149.3 | 11.3 | 0 |
| 152 | 2.08 | + | -0.07 | + |  | 0.9 |  |  | + | 6 | -64.8 | 149.3 | 11.3 | 0 |
| 248 | 2.16 | + | -0.11 | + |  | 1.14 | + | -0.3 | + | 8 | -58.8 | 149.7 | 11.7 | 0 |
| 160 | 2.5 | + | -0.24 | + | + | 0.78 |  |  | + | 8 | -59.9 | 151.8 | 13.9 | 0 |
| 28 | 2.84 | + | -0.38 |  | + | 0.27 |  |  |  | 6 | -66.7 | 153 | 15 | 0 |
| 127 | 2.54 |  | 0.27 | + | + | 0.34 | + | -0.3 |  | 8 | -60.6 | 153.3 | 15.3 | 0 |
| 191 | 2.57 |  | -0.33 | + | + | 0.78 | + |  | + | 8 | -60.7 | 153.4 | 15.4 | 0 |
| 31 | 2.81 |  | -0.33 | + | + | 0.26 |  |  |  | 6 | -67 | 153.7 | 15.7 | 0 |
| 11 | 2.86 |  | -0.23 |  | + |  |  |  |  | 4 | -71.4 | 153.9 | 15.9 | 0 |
| 184 | 2.19 | + | -0.21 | + |  | 0.92 | + |  | + | 7 | -64.6 | 154.3 | 16.4 | 0 |
| 92 | 2.95 | + | -0.43 |  | + | 0.27 |  | -0.16 |  | 7 | -64.7 | 154.5 | 16.5 | 0 |
| 95 | 2.92 |  | -0.4 | + | + | 0.26 |  | -0.17 |  | 7 | -64.8 | 154.8 | 16.8 | 0 |
| 256 | 2.45 | + | 0.05 | + | + | 0.95 | + | -0.38 | + | 10 | -52 | 155.4 | 17.4 | 0 |
| 13 | 2.59 |  |  | + | + |  |  |  |  | 4 | -72.5 | 156.1 | 18.1 | 0 |
| 15 | 2.74 |  | -0.17 | + | + |  |  |  |  | 5 | -70.9 | 156.8 | 18.9 | 0 |
| 63 | 2.61 |  | -0.03 | + | + | 0.3 | + |  |  | 7 | -66 | 157.2 | 19.2 | 0 |
| 29 | 2.56 |  |  | + | + | 0.12 |  |  |  | 5 | -71.3 | 157.7 | 19.7 | 0 |
| 12 | 2.87 | + | -0.24 |  | + |  |  |  |  | 5 | -71.3 | 157.7 | 19.7 | 0 |
| 9 | 2.79 |  |  |  | + |  |  |  |  | 3 | -75.4 | 158.5 | 20.6 | 0 |
| 32 | 2.83 | + | -0.37 | + | + | 0.27 |  |  |  | 7 | -66.7 | 158.6 | 20.6 | 0 |
| 128 | 2.47 | + | 0.32 | + | + | 0.39 | + | -0.29 |  | 9 | -59.2 | 158.9 | 21 | 0 |
| 25 | 2.74 |  |  |  | + | 0.14 |  |  |  | 4 | -74 | 159 | 21 | 0 |
| 14 | 2.59 | + |  | + | + |  |  |  |  | 5 | -72.5 | 160 | 22 | 0 |
| 192 | 2.5 | + | -0.24 | + | + | 0.77 | + |  | + | 9 | -59.9 | 160.3 | 22.4 | 0 |
| 47 | 2.65 |  | -0.03 | + | + |  | + |  |  | 6 | -70.7 | 161 | 23 | 0 |
| 64 | 2.52 | + | 0.05 | + | + | 0.33 | + |  |  | 8 | -64.6 | 161.2 | 23.3 | 0 |
| 96 | 2.92 | + | -0.42 | + | + | 0.26 |  | -0.16 |  | 8 | -64.6 | 161.3 | 23.3 | 0 |
| 16 | 2.75 | + | -0.19 | + | + |  |  |  |  | 6 | -70.8 | 161.3 | 23.3 | 0 |
| 10 | 2.79 | + |  |  | + |  |  |  |  | 4 | -75.1 | 161.4 | 23.4 | 0 |
| 30 | 2.56 | + |  | + | + | 0.12 |  |  |  | 6 | -71.3 | 162.2 | 24.3 | 0 |
| 26 | 2.74 | + |  |  | + | 0.14 |  |  |  | 5 | -73.7 | 162.4 | 24.5 | 0 |
| 48 | 2.62 | + | 0.01 | + | + |  | + |  |  | 7 | -70.4 | 166 | 28 | 0 |
| 19 | 2.29 |  | -0.2 |  |  | 0.21 |  |  |  | 3 | -79.3 | 166.4 | 28.4 | 0 |
| 20 | 2.38 | + | -0.17 |  |  | 0.19 |  |  |  | 4 | -78.2 | 167.5 | 29.5 | 0 |
| 6 | 2.27 | + |  | + |  |  |  |  |  | 3 | -80.4 | 168.5 | 30.5 | 0 |
| 2 | 2.45 | + |  |  |  |  |  |  |  | 2 | -81.9 | 168.6 | 30.6 | 0 |
| 18 | 2.42 | + |  |  |  | 0.13 |  |  |  | 3 | -80.5 | 168.7 | 30.7 | 0 |
| 5 | 2.13 |  |  | + |  |  |  |  |  | 2 | -82.3 | 169.4 | 31.4 | 0 |
| 4 | 2.42 | + | -0.1 |  |  |  |  |  |  | 3 | -80.9 | 169.5 | 31.5 | 0 |
| 21 | 2.14 |  |  | + |  | 0.12 |  |  |  | 3 | -80.9 | 169.6 | 31.6 | 0 |
| 23 | 2.25 |  | -0.18 | + |  | 0.2 |  |  |  | 4 | -79.3 | 169.6 | 31.7 | 0 |
| 22 | 2.27 | + |  | + |  | 0.11 |  |  |  | 4 | -79.3 | 169.7 | 31.7 | 0 |
| 83 | 2.29 |  | -0.21 |  |  | 0.21 |  | -0.02 |  | 4 | -79.3 | 169.7 | 31.7 | 0 |
| 17 | 2.31 |  |  |  |  | 0.15 |  |  |  | 2 | -82.6 | 170.1 | 32.1 | 0 |
| 3 | 2.31 |  | -0.14 |  |  |  |  |  |  | 2 | -82.9 | 170.5 | 32.5 | 0 |
| 84 | 2.39 | + | -0.18 |  |  | 0.18 |  | -0.03 |  | 5 | -78.1 | 171.3 | 33.3 | 0 |
| 24 | 2.35 | + | -0.15 | + |  | 0.18 |  |  |  | 5 | -78.2 | 171.4 | 33.4 | 0 |
| 1 | 2.32 |  |  |  |  |  |  |  |  | 1 | -84.6 | 171.5 | 33.5 | 0 |
| 8 | 2.29 | + | -0.05 | + |  |  |  |  |  | 4 | -80.3 | 171.6 | 33.6 | 0 |
| 7 | 2.17 |  | -0.07 | + |  |  |  |  |  | 3 | -82 | 171.6 | 33.6 | 0 |
| 55 | 2.15 |  | -0.01 | + |  | 0.22 | + |  |  | 5 | -78.7 | 172.4 | 34.5 | 0 |
| 87 | 2.25 |  | -0.19 | + |  | 0.19 |  | -0.02 |  | 5 | -79.2 | 173.5 | 35.5 | 0 |
| 39 | 2.11 |  | 0.02 | + |  |  | + |  |  | 4 | -81.8 | 174.7 | 36.7 | 0 |
| 40 | 2.33 | + | -0.09 | + |  |  | + |  |  | 5 | -80.2 | 175.5 | 37.5 | 0 |
| 88 | 2.36 | + | -0.16 | + |  | 0.17 |  | -0.03 |  | 6 | -78.1 | 175.8 | 37.8 | 0 |
| 56 | 2.29 | + | -0.08 | + |  | 0.19 | + |  |  | 6 | -78.1 | 175.9 | 37.9 | 0 |
| 119 | 2.15 |  | 0 | + |  | 0.21 | + | -0.03 |  | 6 | -78.6 | 176.9 | 38.9 | 0 |
| 120 | 2.29 | + | -0.08 | + |  | 0.18 | + | -0.04 |  | 7 | -78 | 181.2 | 43.2 | 0 |
